# Probing the mutational landscape of the SARS-CoV-2 spike protein via quantum mechanical modeling of crystallographic structures

**DOI:** 10.1093/pnasnexus/pgac180

**Published:** 2022-09-01

**Authors:** Marco Zaccaria, Luigi Genovese, William Dawson, Viviana Cristiglio, Takahito Nakajima, Welkin Johnson, Michael Farzan, Babak Momeni

**Affiliations:** Department of Biology, Boston College, Chestnut Hill, MA 02467, USA; Université Grenoble Alpes, CEA, INAC-MEM, L_Sim, 38000 Grenoble, France; RIKEN Center for Computational Science, 7-1-26, Minatojima-minamimi-machi, Chuo-ku, Kobe, Hyogo 650-0047, Japan; Institute Laue Langevin, 71 Av. des Martyrs, 38000 Grenoble, France; RIKEN Center for Computational Science, 7-1-26, Minatojima-minamimi-machi, Chuo-ku, Kobe, Hyogo 650-0047, Japan; Department of Biology, Boston College, Chestnut Hill, MA 02467, USA; Department of Immunology and Microbiology, The Scripps Research Institute, Jupiter, FL 33458, USA; Department of Biology, Boston College, Chestnut Hill, MA 02467, USA

## Abstract

We employ a recently developed complexity-reduction quantum mechanical (QM-CR) approach, based on complexity reduction of density functional theory calculations, to characterize the interactions of the SARS-CoV-2 spike receptor binding domain (RBD) with ACE2 host receptors and antibodies. QM-CR operates via ab initio identification of individual amino acid residue’s contributions to chemical binding and leads to the identification of the impact of point mutations. Here, we especially focus on the E484K mutation of the viral spike protein. We find that spike residue 484 hinders the spike's binding to the human ACE2 receptor (hACE2). In contrast, the same residue is beneficial in binding to the bat receptor *Rhinolophus macrotis* ACE2 (macACE2). In agreement with empirical evidence, QM-CR shows that the E484K mutation allows the spike to evade categories of neutralizing antibodies like C121 and C144. The simulation also shows how the Delta variant spike binds more strongly to hACE2 compared to the original Wuhan strain, and predicts that a E484K mutation can further improve its binding. Broad agreement between the QM-CR predictions and experimental evidence supports the notion that ab initio modeling has now reached the maturity required to handle large intermolecular interactions central to biological processes.

Significance Statement The threat of emerging pathogens, exemplified by the rapid spread of SARS-CoV-2,has motivated investigations into how pathogens may evolve. In surveying possible evolutionary trajectories, wet-bench screens can only sample a small fraction of possibilities because of practical limitations. Mechanistic modeling can partially overcome these limitations by offering: (1) the flexibility of *in silico* sampling and (2) insights about underlying interaction mechanisms. Here, we employ a complexity reduction quantum mechanical (QM-CR) approach to describes the intermolecular interactions at the amino acid level. Through this approach, we uncover residues critical to spike–receptor and spike–antibody interactions. We find broad agreement between the QM-CR predictions and experimental evidence, showcasing the ability of ab initio modeling to capture biologically relevant intermolecular interactions.

## Introduction

Since SARS-CoV-2 infected the human host, several variants have arisen (1) with distinct changes in the viral spike protein, particularly in the receptor binding domain (RBD). Two trends have been prevalent in the spike evolution: (i) selection toward improved binding to host cells (2) and (ii) selection toward evasion of neutralizing antibodies (nAbs) (3–6). Anticipating the evolutionary trajectory of viruses is a long-established relevant topic in the scientific community (7). Presently, the main approach in this direction is high-throughput in vitro screening of mutants [e.g. (8, 9)]; however, such an approach does not directly identify the mechanisms that make a given mutation more, or less, beneficial. In this work, we show how the recent developments in ab initio modeling can complement experimental results and offer detailed mechanistic insights.

Traditionally, full quantum mechanical (QM) models of intermolecular interactions are only employed for small molecules of about a hundred atoms (10, 11); larger molecules have proven computationally challenging for full QM investigations. Nevertheless, in silico approaches alternative to full QM have been successful. Molecular docking (12–14), relying on geometrical constraints to assess intermolecular interactions, has been used to survey small-molecule candidates in drug discovery (15). Force-fields (FFs) have also been successful (16, 17), whenever previous adequate parameterization is available (18). Hybrid quantum mechanics/molecular mechanics (QM/MM) methods are also common in describing enzyme–substrate systems (19), and have been successfully applied to SARS-CoV-2 (20–30). QM/MM uses QM simulations for a small portion of the system (tens of atoms) (31), leaving the remaining regions to be modeled with a less computationally demanding MM simulation, driven by FFs.

To mechanistically characterize SARS-CoV-2 spike–receptor and spike–nAb interactions, we apply a recently developed approach for large-scale electronic structure calculations: complexity reduction in density functional theory (DFT) calculations (32, 33), hereafter called QM-CR. QM-CR differs from previous approaches in requiring no targeted parameterization or prior knowledge about the nature or sites of interactions, and it is based on full QM calculations on the entire system. QM-CR leverages recent progress in computational chemistry (34) to handle tens of thousands of atoms in a single simulation. This enables us to capture and investigate biological processes involving several hundreds of amino acids, including the SARS-CoV-2 spike interactions. Recent efforts on SARS-CoV-2 have generated structural and biochemical data that can be used to validate QM-CR predictions. In particular, the high level of detail from recent contributions gives us new insight to complement experimental data or analysis based on regression models (35–38).

Importantly, QM-CR can reveal the mechanisms behind intermolecular binding by decomposing interactions into chemical/short-ranged (which imply a shared electron) versus electrostatic/long-ranged (which do not involve shared electrons). We define as “hotspots” amino acids with a significant chemical contribution to the intermolecular interactions. To further investigate the contribution of individual amino acids, single point mutations can be introduced into a protein's (e.g. the spike) primary structure. We employ the BigDFT computer program (39), based on an ab initio DFT approach on a set of fully atomistic 3D structural models, to simulate intermolecular interactions of interest with a computational cost manageable on modern supercomputers.

In this work, we focus our analysis on the E484K mutation for three main reasons. First, our analysis identifies residue E484 as the main interface weak link in the interaction of the SARS-CoV-2 Wuhan strain with the human receptor ACE2 (hACE2); conversely, the same residue is beneficial to binding the bat *Rhinolophus macrotis’* ACE2 (macACE2). Second, we show that an E484K mutation alone can disrupt the neutralizing effect of specific antibodies. In addition, we also highlight the strong modular character of the E484K mutation and show that, if imposed on existing SARS-CoV-2 variants such as Delta, it can enhance binding to hACE2, potentially identifying future viral evolutionary trajectories. Finally, we argue that ab initio models are now at the point of providing mechanistic insights on molecular interactions central to biological processes.

## Results

We focus our analysis on the impact of the E484K mutation on antibody evasion and cellular receptor binding. Prior experimental and computational data have shown that spike variants with the E484K mutation in the RBD can evade antibodies C144 and C121 (38, 40). E484K is also a typical signature mutation of the RBD of the Gamma and Beta variants. We test our QM model as an agnostic predictor to explain the interaction of the viral spike (the original Wuhan version or the E484K-mutated one) with host receptors and nAbs.

### QM-CR underscores hotspots of spike–hACE2 interactions

**Fig. 1.**
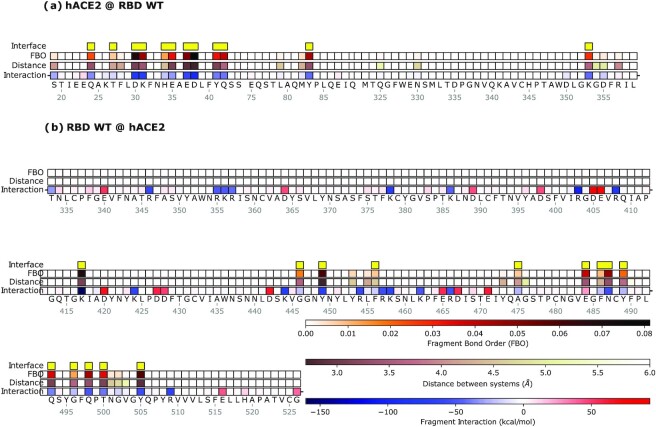
Mechanistic characterization of the binding between Wuhan strain's spike and hACE2. Data are plotted on the sequence of hACE2 (a) and the spike RBD (b). Letters represent single amino acid residues; yellow bars indicate interface residues, identified with the FBO threshold. “FBO” is the Fragment Bond Order values, and “Distance” is the distance of a residue to the nearest atom of its ligand. “Interaction” is the chemical/electrostatic force shown as attractive (blue) or repulsive (red), with darker colors indicating stronger effects.

We examine the interaction between the Wuhan-type (WT) spike RBD and hACE2 as its native substrate. In this analysis, we calculate the overall effect of each amino acid residue on its respective interactor, either on the spike side or on the hACE2 side; the contribution to the binding energy can either be attractive/stabilizing or be repulsive/de-stabilizing (Fig. 1).

We use the Fragment Bond Order (FBO) (32), calculated using the electronic structure of the system in proximity of a given residue, as a measure of the strength of the interaction in the proximity of the interface between the two interacting molecules (Table 1). In Fig. 1, we have highlighted residues with large FBO as well as those close to the geometric interface. Residues with both large FBO and interface proximity are determined as major contributors to the intermolecular interactions. This analysis reveals the contribution of each residue to the overall binding performance, highlighting which amino acids facilitate or hinder binding, and how. In the following sections, we use FBO to draw an interaction network of the interface to detail the chemical interactions among residues and their stabilizing or destabilizing role. Details of the procedure are provided in the Supplementary Material (“Details of the fragmentation procedure”). As an alternative visualization, the contribution of each amino acid residue to the binding can also be highlighted over the 3D physical arrangement of the two molecules (Fig. S1).

**Table 1.** Prospectus of the main concepts and quantities constituting the model.

**Electron density**	The distribution of electrons in a given molecular system. The electron density determines the nature and strength of the chemical bonds between interacting molecules. Such an “electron cloud" is the main emerging property of the underlying atomic structure in defining the chemical characteristics of a molecule.
**Fragment**	The modular elements into which the electron cloud can be partitioned, for example, an amino acid. The model partitions the electron cloud into physically consistent regions and/or verifies the consistency of a pre-defined partitioning; every such region is defined as a fragment.
**FBO**	The descriptor of the inter-fragment interactions. FBO is the main quantity used in the model to represent the connection pattern of the fragments of interacting molecules.
**Fragment interactions**	From the results of the model and the features of the fragments it is then possible to calculate the interaction strength between any two fragments. Such interaction has both a chemical/short-range term that is always attractive, and an electrostatic/long-range term that can be attractive or repulsive.
**Final output**	At the end of the simulation, BigDFT provides a simple representation of the strength of interaction between fragments of the two molecules. The model can describe the energy and nature of the acting chemical bonds. This enables a mechanistic explanation and/or prediction of how specific amino acid substitutions or deletions, in spikes or nAbs, impact the interactions with their hACE2 substrate or the viral spike, respectively.
**Hardware requirements**	The model requires massively parallel calculations via high performance computing. On a modern supercomputer, hundreds of simulations can be performed in a time frame of one hour.

All the elements here discussed are general and therefore applicable, without previous parameterization, to any given set of atoms for which atomistic structural representations are available.

### QM-CR identifies the spike E484 residue as the weak link in the binding to the host receptor hACE2

**Fig. 2.**
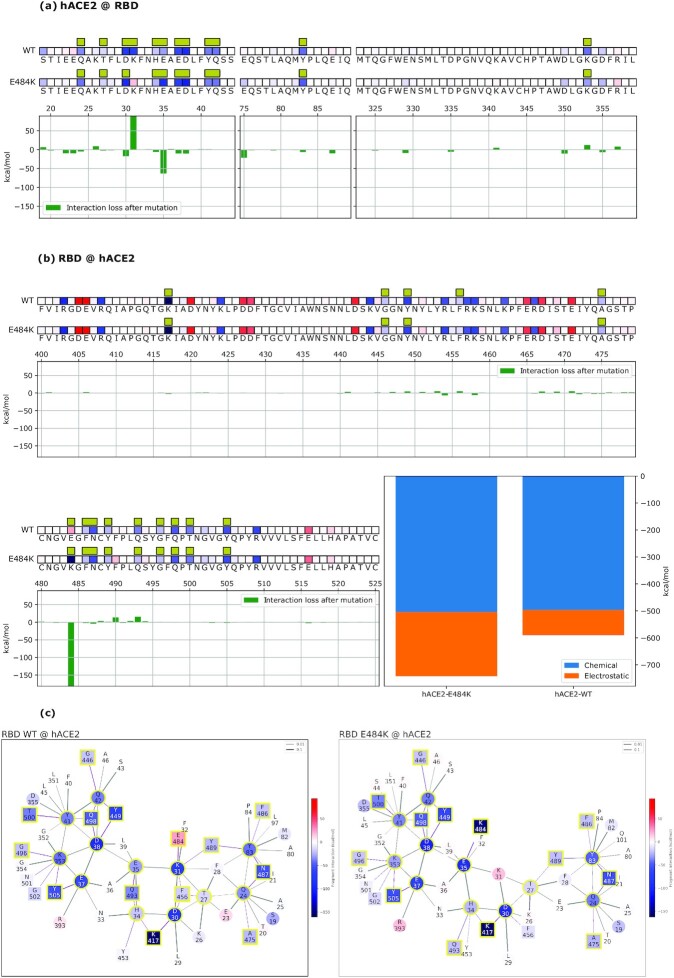
Data are plotted on hACE2 (a) and on the Wuhan spike (b) primary structure bound to the Wuhan spike (WT) and the mutated one (E484K). Amino acids are represented by the corresponding letters and numbered on the histogram's horizontal axis. Interface residues are highlighted by yellow bars and their overall effect on the other molecule is indicated by red (repulsive) or blue (attractive) tiles. Histograms underneath the sequences show the relative change in binding energy of the E484K mutated variant relative to the Wuhan strain, with positive and negative values indicating weaker and stronger binding, respectively. Bottom right histograms represent the overall binding energy of hACE2 with the Wuhan spike versus the mutated one, partitioned into chemical and electrostatic contributions. Interaction networks (Wuhan spike-hACE2 to the left, and mutated spike-hACE2 to the right), including FBO-interface residues and their coordinated interactors are shown (c). Squares depict spike residues and circles depict hACE2 residues, with red color for repulsive and blue color for attractive energy. Yellow outlines highlight interface residues. Bonds are purple when intermolecular or black when intramolecular.

FBO values pinpoint the hotspots of the RBD-hACE2 system (Fig. 2). On the hACE2 side (Fig. 2a), Q24, T27, D30, K31, H34, E35, E37, D38, Y41, Q42, Y83, and K353 stand out, in agreement with known data (41). On the spike side (Fig. 2b), a more diverse layout emerges, on and off the interface, with several residues displaying repulsion. However, residue E484 shows the unique trait of being simultaneously repulsive and at the interface with hACE2, via a short-range interaction with the K31 residue (Fig. 2c). Since the chemical interaction is intrinsically attractive, the overall repulsive interaction indicates that another residue in the vicinity cancels the chemical attraction with an electrostatic repulsion. Overall, in the WT structure, E484 destabilizes the binding to hACE2. From this analysis, we conclude that the Wuhan spike RBD harbors a suboptimal residue at the 484 position for hACE2 binding.

**Fig. 3.**
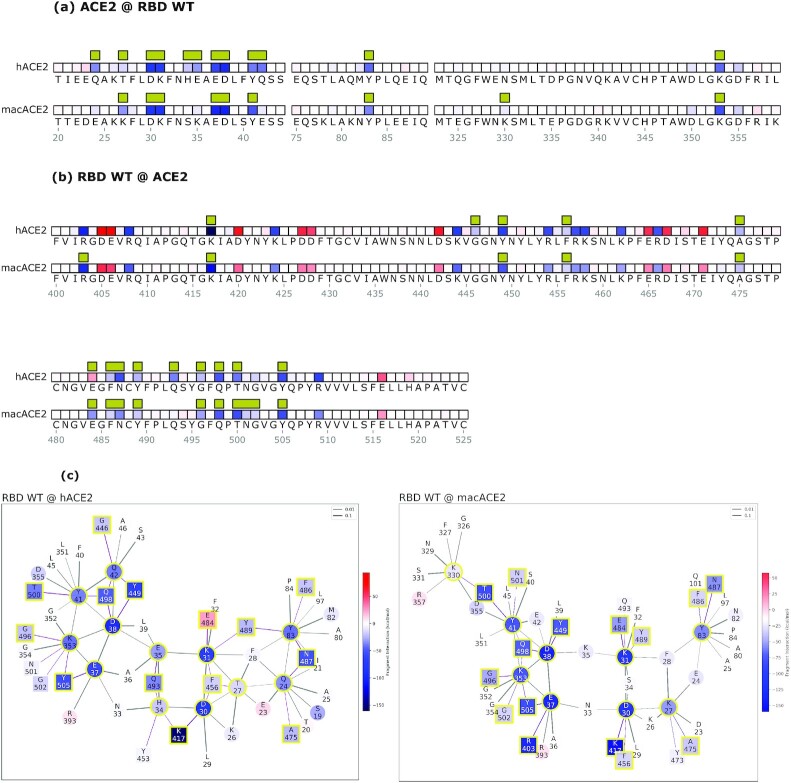
Mechanistic characterization of the Wuhan spike binding to the human ACE2 (hACE2) and *R. macrotis* ACE2 (macACE2). Data are plotted on the ACE2 primary structure (a), and on the Wuhan spike RBD (b), when binding to the human (hACE2) and the bat (macACE2) receptor. Amino acid residues are labeled with letters and numbered. Interface residues are highlighted with a yellow bar, red tiles are repulsive residues, and blue tiles are attractive residues; see the rest of the figure for energy scales. The interaction networks (c) represent the hACE2-spike system on the left, and macACE2-spike on the right; circles are ACE2 residues, squares are spike residues. Interface residues are highlighted with a yellow bar, red tiles are repulsive residues, and blue tiles are attractive residues. Bonds are purple when intermolecular or black when intramolecular, and their thickness represents the strength of the FBO between residues.

To further investigate the impact of E484, we tested the model on the available 3D crystal structure of the human homologous ACE2 receptor in *R. macrotis*, a host species with a more adapted SARS-CoV-2 interaction (41). In this simulation (Fig. 3a-b, second rows), the E484 residue is instrumental to the binding by being strongly attractive to the *R. macrotis* ACE2 (macACE2). Notably, in both hACE2 and macACE2, the interactor with E484 is the ACE2 residue K31. This means that the macACE2 sequence has residues, proximal to the K31 hotspot, that exert an attractive electrostatic force on E484. A closer inspection of the two sequences reveals that this attractive force comes from the K35 residue, which in hACE2 is replaced by glutamic acid. Thus, the model highlights a stark contrast between human and bat receptors.

We further confirmed the role of E484 by introducing the E484K mutation into the viral spike and then assessing the interaction with hACE2 (Fig. 2). The E484K mutation improves the spike–hACE2 binding energy by about 32% (Fig. 2b, histogram), switching the main hACE2 interacting residue from K31 to E35. Such an interaction, driven by electrostatics, represents a net improvement of the Wuhan-hACE2 network. Conversely, the same mutation does not affect the spike binding energy to macACE2 in the same position, where the bat receptor hosts a lysine. In other terms, for macACE2, K484 clearly does not engage K35, and would actually disappear from the interface (Fig. S2). The resulting interaction network is rearranged, and the interface binding energy is not improved by the mutation. Therefore, the model shows a more functional interaction between macACE2 and Wuhan RBD, possibly the result of a longer adaptation by SARS-CoV-2 to *R. macrotis*, compared to the human receptor. In the hACE2 interaction, the E484 spike residue belongs to a suboptimal sector of the chemical interface, suggesting that other RBD adaptations in this sector are likely to improve the binding.

### QM-CR shows how nAb C121 loses binding to the E484K mutated spike

**Fig. 4.**
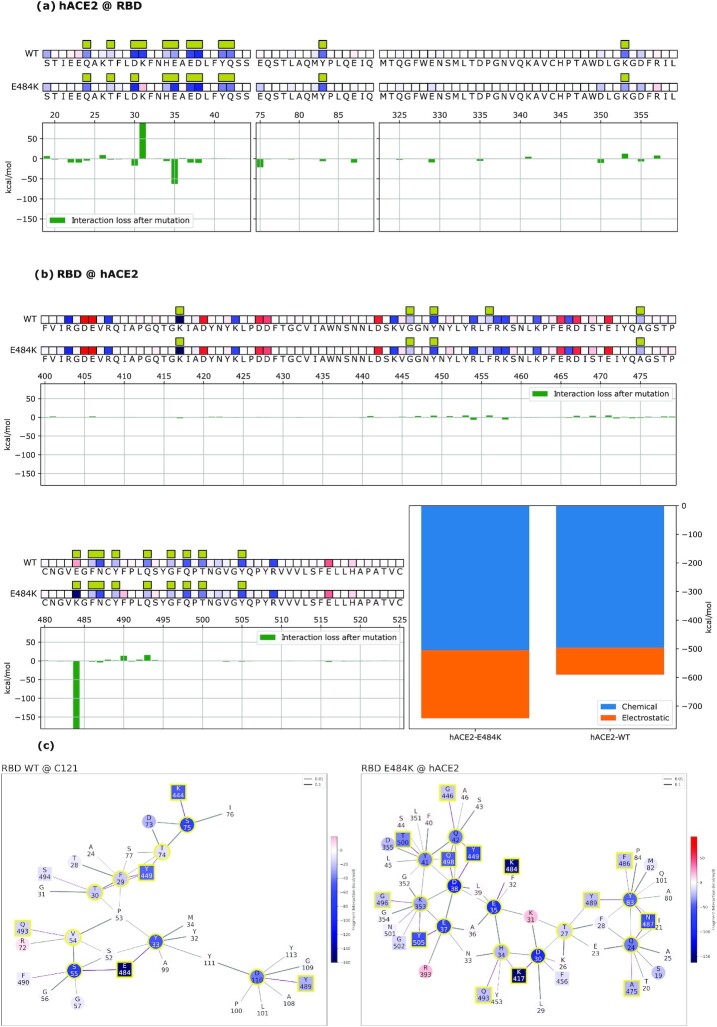
Mechanistic characterization of C121 binding to the Wuhan strain spike protein and energetic changes as a result of the E484K spike mutation. Data are plotted on the spike primary structure (a) and on C121’s Heavy-Chain (b) considering the different bindings via the Wuhan spike (WT) and the mutated one (E484K). Amino acids are represented by letters and numbered on the histogram's horizontal axis. Histograms underneath the sequences represent the relative change in binding energy of the second row relative to the first one (Wuhan strain). The bottom right histograms represent the overall binding energy of C121 with the Wuhan spike (left) and the mutated one (right) and its characterization as chemical or electrostatic. The row above each sequence shows the chemical or electrostatic forces as attractive (blue) or repulsive (red), with darker colors indicating stronger effects. Interaction networks with C121 nAbs are shown (c). Network nodes are represented in red (repulsive) or blue (attractive) based on their effect on their counterparts. Residues at the binding interface are highlighted by a yellow outline. Bonds are plotted as purple when intermolecular or black when intramolecular and their thickness is related to the strength of the FBO between residues.

We identify the hotspots between the Wuhan spike RBD and C121 nAbs (Fig. 4 and Fig. S1) (see results for C144 nAb in Fig. S3). Residue E484 is the main spike interactor with C121 nAb. Other relevant sites are residues K444, Y449, F486, Y489, and Q493. On the C121 side, residues Y33, S55, and S75 are pivotal for the Wuhan spike binding. The model estimates that among all the residues contributing to the interaction, the individual contribution of E484 amounts to around 50% of the total. The interaction network (Fig. 4c) shows E484’s binding to residues Y33 and S55 of C121. Once the E484K mutation is imposed, we observe a rearrangement of the interaction network, and a substantially lower binding energy between the spike and the antibody. Specifically, E484K reduces the connectivity at the 484 residue in the interaction network and modifies the interactions on the C121 side toward decreased stability. Only the S52 residue is stabilized by the mutation, but not to the point of compensating for the loss of attraction at other residues. Overall, once the mutation is applied, we observe a substantial decrease of about 25% of the total binding energy, largely because of reduced short-range interactions. The model concludes, with no a priori information other than the experimental crystal structure, that the E484 residue is the essential actor in the binding by nAb C121, and that a targeted point mutation will substantially affect said binding. The analysis of C144 nAb shows comparable results. Moreover, C144 undergoes a substantial rearrangement of its interaction network in response to the mutation, arguably a consequence of the original higher connectivity of the residue E484 in the binding, compared to the C121 case: five C144 residues are involved (Y51, S52, G53, G54, and S55) compared to two C121 residues (Y33 and S55) (Fig. S3). Interestingly, the importance of E484 also appeared in previous a work in which E484 mutants arise under the selective pressure of nAbs (42).

### QM-CR predicts that the E484K mutation strengthens the binding of the Delta spike to hACE2

Starting from the Wuhan strain crystal structure, we generate a virtual crystal structure to represent Delta (B.1.617.2) in conjunction with hACE2 by substituting its characterizing RBD mutations (L452R and T478K) into the Wuhan spike crystal structure. Such residue mutations belong to an off-interface sector of the RBD (see Fig. 1). Our simulations identify the same FBO interface residues found for the Wuhan strain. However, differently from the other tested interaction networks, a substantial contribution to the overall binding energy of Delta to hACE2 comes from off-interface residues via their long-range electrostatic effect on their counterparts, highlighting the relevance of including residues beyond the interface region in the analysis of binding.

**Fig. 5.**
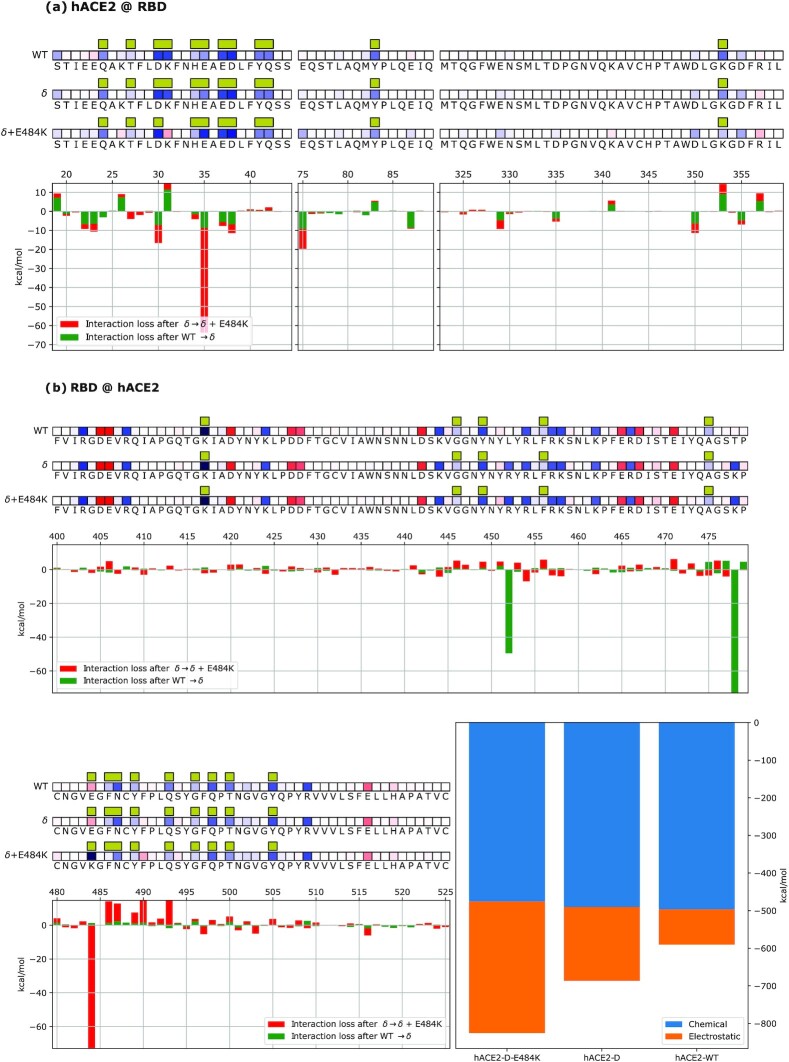
Mechanistic characterization of spike–hACE2 binding suggests that Delta + E484K spike has stronger hACE2 binding than the Delta variant. (a) Data are plotted on hACE2 primary structure bound to the Wuhan spike (WT), Delta spike (*δ*) and Delta + 484 K spike (*δ* + 484 K). Amino acids are represented by the corresponding letters and numbered on the histogram's horizontal axis. Interface residues are highlighted by yellow bars and their overall effect on the other molecule is indicated by red (repulsive) or blue (attractive) squares (energy scale is identical to the one employed in the other figures). Histograms underneath the sequences show the relative change in binding energy (green: Delta compared to Wuhan; red: Delta + E484K compared to Delta). (b) Data are plotted on the viral spike primary structure bound to the Wuhan spike (WT), Delta spike (*δ*), and Delta + 484 K spike (*δ* + 484 K). Amino acids are represented by the corresponding letters and numbered on the histogram's horizontal axis. Interface residues are highlighted by yellow bars and their overall effect on the other molecule is indicated by red (repulsive) or blue (attractive) squares (energy scale is identical to the one employed in the other figures). Histograms underneath the sequences show the relative change in binding energy (green: Delta compared to Wuhan; red: Delta + E484K compared to Delta). Bar plots on the bottom right represent the overall binding energy of hACE2 with the Wuhan, Delta, and Delta + E484K strains, partitioned into chemical or electrostatic contributions.

Furthermore, when testing the binding of the Delta–hACE2 system after introducing the E484K mutation, the simulation shows that E484K is compatible with the Delta variant and further strengthens the overall binding to hACE2. This in silico*-*generated variant, solely based on theoretical grounds, displays a stronger binding to hACE2 than either E484K or Delta variants individually (Fig. 5).

## Discussion

Recently developed complexity reduction tools in DFT calculations have allowed full QM simulations of systems with several thousands of atoms. These advances have bridged the gap that had so far hindered full QM ab initio modeling of larger molecules that are often of interest in biology. A computational approach that can capture biologically relevant intermolecular interactions, such as protein–protein interactions, has untapped potential for better mechanistic understanding of biological phenomena at a molecular level.

In this work, we use the BigDFT code to implement an ab initio QM simulation of the electronic properties of a given set of atoms as large as a full protein–protein system. Through this model, we decompose the interaction between two biological macro-molecules, spike RBD and receptor/antibody, into the individual energetic contributions of each of the amino acid residues involved. Additionally, the model characterizes the nature of these contributions into two main categories: (1) short-range/chemical and (2) long-range/electrostatic. Ultimately, we infer a network of interactions with amino acid residues of the two interacting molecules as nodes, and the inter-residue binding strength as edges. This interaction network is based on the electronic structure of the protein–protein system.

We focus on the viral spike interaction with ACE2 as its natural receptor, and with nAbs C121 and C144. We demonstrate that a QM model, assessing the interactions among the residues of an intermolecular biological system, enables mechanistic insight into how SARS-CoV-2 interacts with its host. The QM-CR model identifies the E484 residue as the only interface element hindering the binding between the Wuhan strain and hACE2, making it the most evident weak link of the Wuhan spike binding to the human host. The E484K mutation is shown by the model as a direct solution to this hindrance by improving binding to hACE2 and presumably constituting an evolutionary advantage, as supported by its emergence among several successful variants. Interestingly, QM-CR also shows that the E484 residue stabilizes the interaction between the Wuhan viral spike and the bat receptor macACE2 from *R. macrotis*. We interpret this as an indication that the Wuhan strain is better adapted to a bat-like ACE2, and the rise of changes at E484 constitutes an adaptation specific to the human host.

In agreement with known data, QM-CR predicts loss of interaction between the SARS-CoV-2 spike and nAbs C121 and C144 once the E484K mutation is imposed on the spike of the Wuhan strain. The RBD residue E484 emerges as the main and fundamental spike fragment enabling the binding event, and therefore neutralization. These data suggest that nAbs challenging the spike at E484—the very residue that most hinders hACE2 interaction—provide an ulterior selective pressure for the virus to find alternatives to the original phenotype at this position.

By analyzing the competition between short- and long-range interaction contributions, we have shown that, compared to the Wuhan strain, the charge-shift E484K mutation substantially increased (by about 30%) the binding energy to hACE2. On the RBD side, the model also highlights how the effect of E484K is focused on the 484 position, with limited off-target repercussions for the spike's binding (Fig. 2). We argue that this trait qualifies the E484K mutation as highly “RBD-modular" and readily achievable in an already well-adapted spike structure. The contribution of E484K to the binding is largely long-range/electrostatic, therefore less dependent on a specific steric conformation. Our simulations are motivated by the available empirical data in identifying the E484K mutation as a particularly likely evolutionary outcome, based on increased SARS-CoV-2 infectivity and antibody evasion. We thus examined the potential impact of the E484K mutation on spike–hACE2 binding in the background of the Delta variant. Our model suggests that E484K affects spike–hACE2 and spike–nAb binding in a modular fashion. Thus, if acquired by the Delta strain, E484K further increases binding, possibly contributing to increased infectivity. We acknowledge that infectivity is a multifactor process, of which receptor binding is only one among multiple actors.

Our investigation is focused on characterizing individual amino acid contributions to the different performances of alternative spike structures in binding hACE2, especially to assess the hypothetical relevance of present and future single point mutations imposed on available crystal structures. Binding to ACE2 is the first step for SARS-CoV-2 infection, and is therefore central to the overall fitness of a given viral variant. In the context of viral evolution toward improved human ACE2 binding, we intend to identify the structural traits that represent the objects of selection; when compared to the closest experimental dataset available (9), the quantities we compute provide QM simulations, which largely align with empirical results (Fig. S6) (43, 44).

The QM-CR approach is performed on all-atom in silico structures as inputs. In this context, we have applied the QM-CR method to crystal structures available in the PDB database, as well as variations of them, whenever crystalized structures are unavailable. Our analysis does not take into account conformational changes (which recent work has shown take place on the order of microseconds for spike–hACE2 interactions (45, 46)), conformational changes would require applying QM-CR to a population of structures coming from, for instance, subsampled Molecular Dynamics (MD) trajectories (47). Furthermore, due to the nature of the QM-CR analysis and the use of a single frame, interaction energies do not account for entropic effects or rearrangement (electronic or nuclear) after disassociation. Interactions have also been partitioned into per-amino-acid contributions, which introduces some error terms; however, this can be controlled using measures provided by the QM-CR methodology (see Supplementary Material, “Details of fragmentation procedure”). For this study, i.e. the case of E484K, the model's predictions align with available empirical data even when using the initial virtual crystal structures. In this specific case, this may be due to the long-range impact of E484K as a charge-shift mutation. Moreover, in the vicinity of the interface, the QM-CR approach produces an interaction network, which at the very least encodes the first-order effects that a mutation can induce in the chemical bonds of the interface.

The crystal structures employed for the Delta spike variant are not associated to an experimental result. They are virtual approximations, obtained via local energy minimization. The approximation assumes that no major structural changes from the reference Wuhan spike occur when single point mutations are introduced. In the Supplementary Material (Fig. S4), we show evidence that such an approximation is reasonable, at least for the combination of mutations characterizing the Beta variant RBD: E484K, N501Y, and K417N. We employ a well-established DFT approximation, PBE + D3, which provides reliable information on coarse-grained quantities and trends (32, 48), and simulates structures in their relaxed positions (49). Overall, we deem our method to be a balanced compromise between accuracy and modeling complexity.

The maturity of large-scale QM calculations represents a unique opportunity to employ full QM approaches to uncover the interaction mechanisms. Such mechanisms are presently inaccessible to other, more conventional computational approaches. We also show that an ab initio modeling in QM-CR provides insights useful for comparison with experimental data, supporting its capability to offer predictive power for intermolecular interactions of biological relevance. Finally, we argue that QM-CR can be correlated to high throughput calculations of libraries of mutated structures aimed at identifying potential antibody escape routes for SARS-CoV-2 and, being unbiased and agnostic, can be readily applied to other biological systems.

## Methods

### Computational approach

We perform a full QM model, as implemented in the BigDFT computer program suite (50). The approach employs the formalism of Daubechies wavelets to express the electronic structure of the assemblies in the framework of the Kohn–Sham (KS) formalism of DFT (39). The electronic structure is expressed, by both the density matrix and the Hamiltonian operator, in an underlying basis set of support functions—a set of localized functions adapted to the chemical environment of the system. Such functions are expressed in Daubechies wavelets, typically using one to four support functions per atom as the basis set. The electronic density matrices, as well as the Hamiltonian expressed in the BigDFT basis set, are analyzed to provide quantum observables of the systems. The code provides efficient and accurate QM results for full systems of large sizes, delivering excellent performance on massively parallel supercomputers. In the present study, we employ the PBE approximation corrected by dispersion D3 correction terms (51) and Hartwigsen-Goedecker-Hutter (HGH) pseudopotentials (52). The CheSS library (53) has been employed to calculate the system's density matrix. A comparison of the inclusion of an implicit solvent, with respect to gas phase calculations shows that interaction energies at interface residues are only marginally affected by the presence of the solvent (Fig. S5).

Each calculation includes approximately 12,000 atoms and requires about 2 h of wall time on 32 compute nodes of the IRENE-Rome supercomputer, at the TGCC supercomputing center in Saclay (Paris, France). A similar approach has been previously used, in conjunction with the other atomistic techniques described in the introduction, to investigate the interaction patterns of the SARS-CoV-2 main protease with natural peptidic substrates, and to design peptide inhibitors tested in vitro (47).

### Procedure

Starting from a representative 3D model of the molecules as our input, we calculate the system's electronic structure, from which we extract various quantities. We draw a contact network to identify relevant chemical interactions among the spike RBD and the various interactors considered in this study. The strength of the inter-residue interaction is quantified by the FBO (54), calculated using the electronic structure of the system in proximity of a given residue. Such an approach has been previously described in detail (39, 55) and is summarized in Table 1.

We use the FBO to identify the interface residues, defined as the amino acids of the counter-ligand that have a non-negligible value, above a set threshold of the FBO, with the ligand. In contrast to a simple geometrical indicator like the RBD-ligand distance, the FBO provides a metric that enables a nonempirical identification of steric hotspot interactions. We here identify as chemical hotspot interface residues the amino acids, which exhibit a FBO value with the ligand larger than 7 × 10^–3^. Such a threshold is obtained by comparing the hydrogen bonding interaction network of the SARS-CoV-2 main protease to its natural peptidic substrates, derived from traditional FF analysis and the equivalent FBO network (47).

Once the chemical connection among amino acids is identified, we assign to each residue its contribution to the binding interaction between the two subsystems. We calculate these interaction terms from the output of the DFT code and interpret them as two parts. First, a long-range electrostatic attraction/repulsion term, defined from the electron distributions of each of the fragments (even when far apart, two fragments may still interact). The remaining term, which can only be attractive, is provided by the chemical binding between the fragments, and is nonzero only if the electronic clouds of the fragments superimpose (short-range). This term is correlated with the FBO strength, and we identify it as the chemical interaction.

By including long-range electrostatic terms, the decomposition enables us to single out relevant residues not necessarily residing at the interface. In this way, the model provides an ab initio representation of the RBD-ligand interactions as the final output.

### Crystal structures and generation of mutant virtual structures

Crystallographic structures are obtained from the RCSB database (56) using PDB entries 6M0J (hACE2), 7K8X (nAb C121), 7K90 (nAb C144), and 7C8J (macACE2). Protonation of histidines and other titratable residues is assigned a pH of 7, based on the PDBFixer tool in OpenMM (57, 58).

Virtual structures are generated by imposing point mutations on the original structure. Structure relaxations are performed by optimizing the crystal geometry with the OpenMM package using the AMBER FF14SB force field (59). While such optimized structures do not represent the full panorama of conformations that might exist at a finite temperature, the resulting structures are interpreted as one plausible representative among the possible conformations of the system. To further verify this statement, we compared the difference in the interaction pattern obtained from the experimental crystal structure of the Beta variant in conjunction with hACE2 (PDB 7VX4) to the same quantity from the combined action of each point mutation characterizing the Beta RBD (E484K, N501Y, and K417N), applied on virtual crystals derived from WT-RBD (6M0J). We verify (see Supplementary Information) that the interaction difference on the RBD of the two real crystals corresponds to the overall sum of the contributions of each of the point mutations. This fact, on the one hand, confirms the modular impact of each point mutation to the overall binding, on the other hand, suggests that the impact due to conformational rearrangements is of higher order, for this variant.

## Supplementary Material

pgac180_Supplemental_File

## Data Availability

All data are included in the manuscript and/or Supplemenatry Material.
