# Supplementary material for: Probing the mutational landscape of the SARS-CoV-2 spike protein via quantum mechanical modeling of crystallographic structures
Source: PNAS Nexus. 2022 Sep 1;1(5):pgac180. doi: 10.1093/pnasnexus/pgac180 (PMC9802038; doi:10.1093/pnasnexus/pgac180)
Supplement: pgac180_Supplemental_File [file pgac180_supplemental_file.pdf]

Supplementary Material

3D representations of QM-CR predictions on the Wuhan spike and its variants

A) WT at ACE2

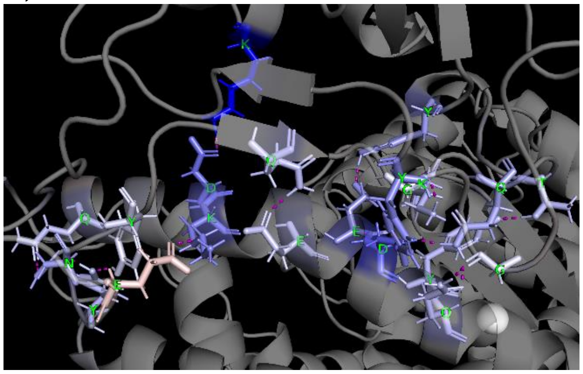

B) E484K ACE2

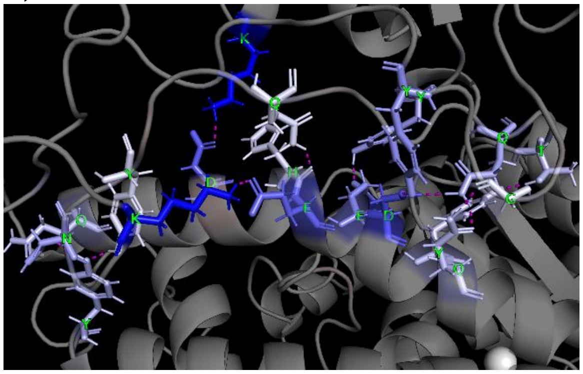

C) WT at macACE2

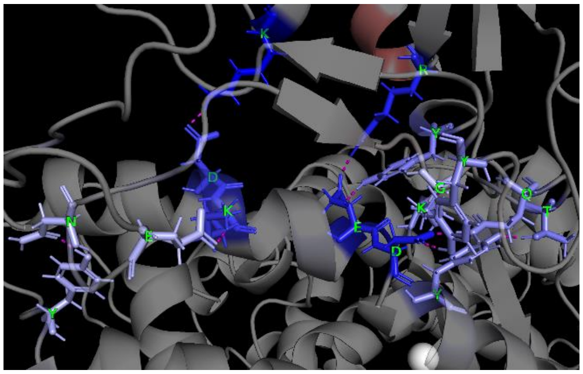

D) WT C121

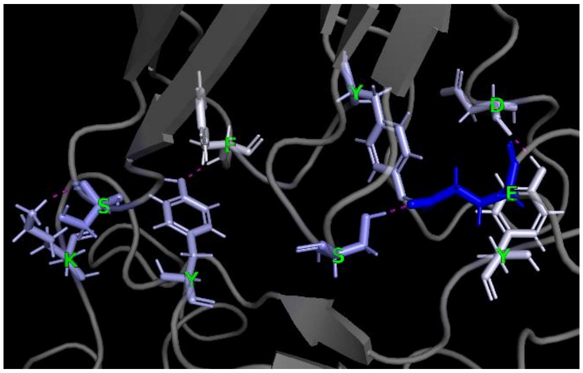

E) E484K at C121

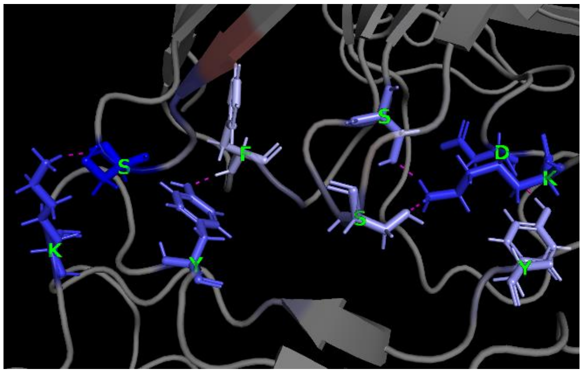

Fig S1. The color code of structural regions highlighted in hues of blue and red matches the one used for the binding characterizations and the interaction network, i.e. blue/attractive and red/repulsive. The letters represent relevant amino acids.

QM-CR shows how the E484K spike does not improve the binding energy to R. macrotis ACE2 receptor (macACE2), compared to the Wuhan strain

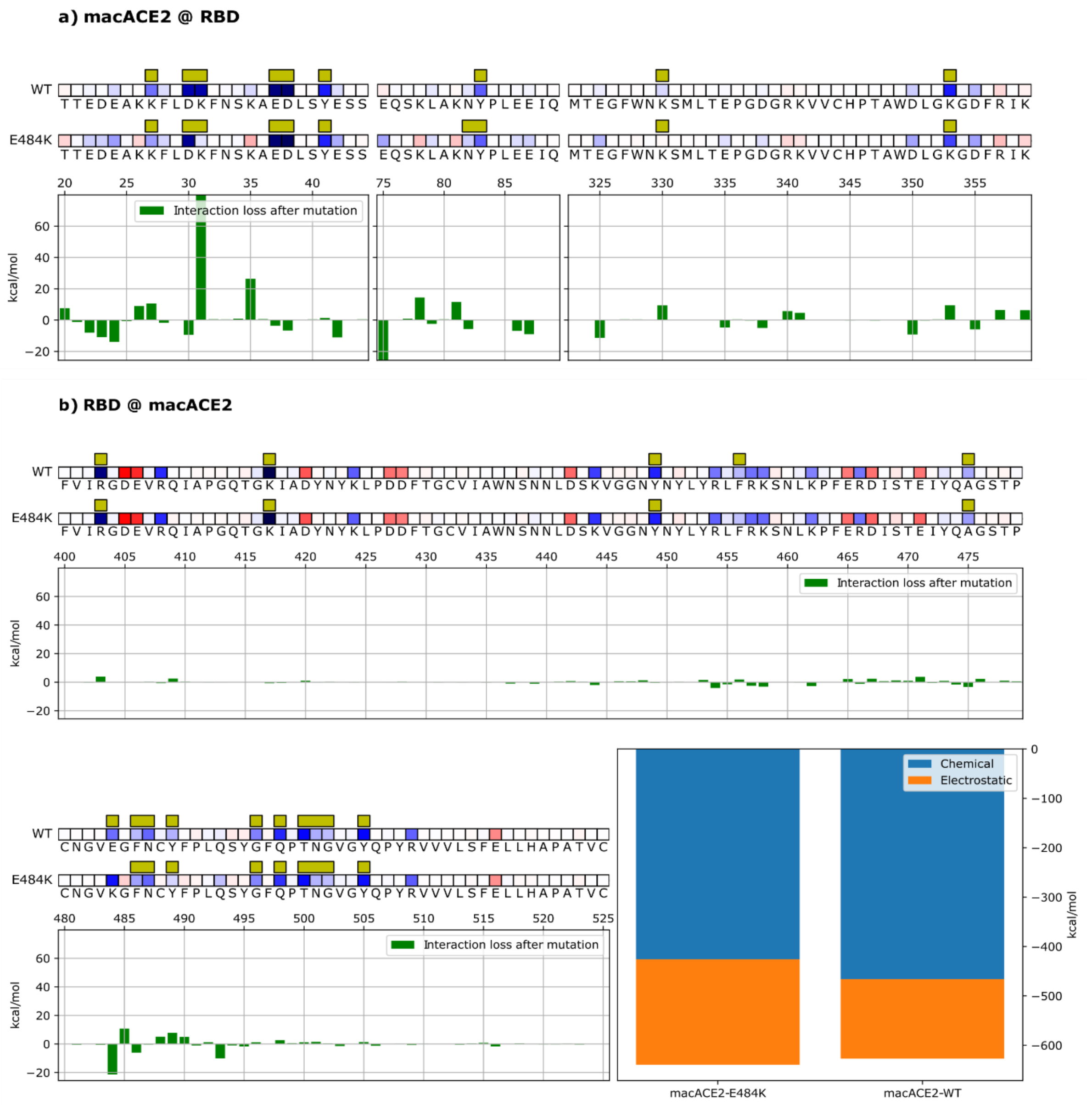

Fig S2. Mechanistic characterization of Wuhan and mutated (E484K) spike binding to macACE2. Data are plotted on the macACE2 primary structure (panel a) and on Spike RBD (panel b), considering the different bindings to the Wuhan spike (WT) and the mutated one (E484K). Amino acids are represented by the letters and numbered on the histogram's horizontal axis. Histograms underneath the sequences represent the relative change in binding energy of the E484 mutated strain relative to the Wuhan strain. The bar plots (bottom right) represent the chemical and electrostatic contributions in the binding of macACE2 to the Wuhan spike and the mutated one.

c)

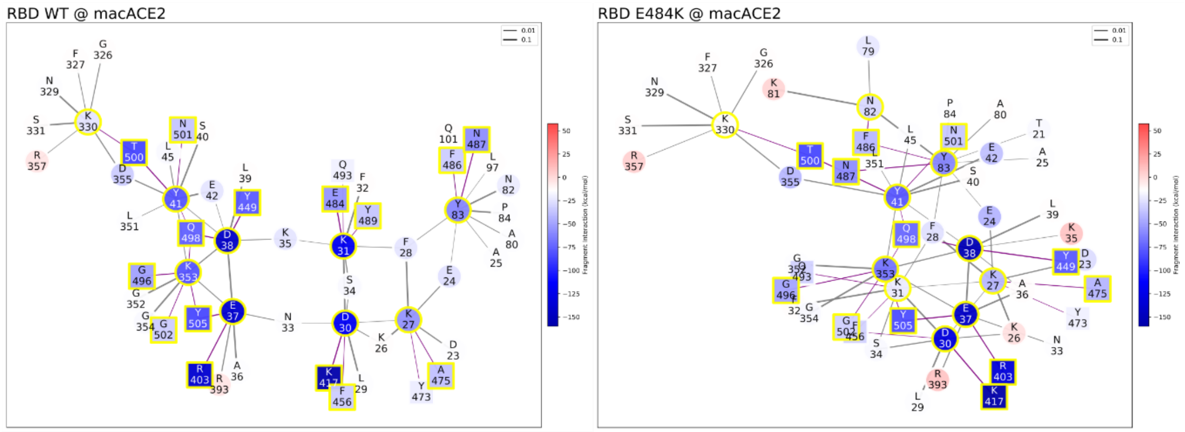

Fig S2 (cont.). Interaction networks (RBD WT @ macACE2 to the left, RBD E484K @ macACE2 to the right) Bonds are purple when intermolecular or black when intramolecular, their width being related to the strength of the FBO between residues. Graph nodes are represented in red (repulsive) or blue (attractive) based on their effect to their counterpart and highlighted by yellow squares when at the binding interface.

QM-CR shows how nAb C144 loses binding to the E484K mutated spike

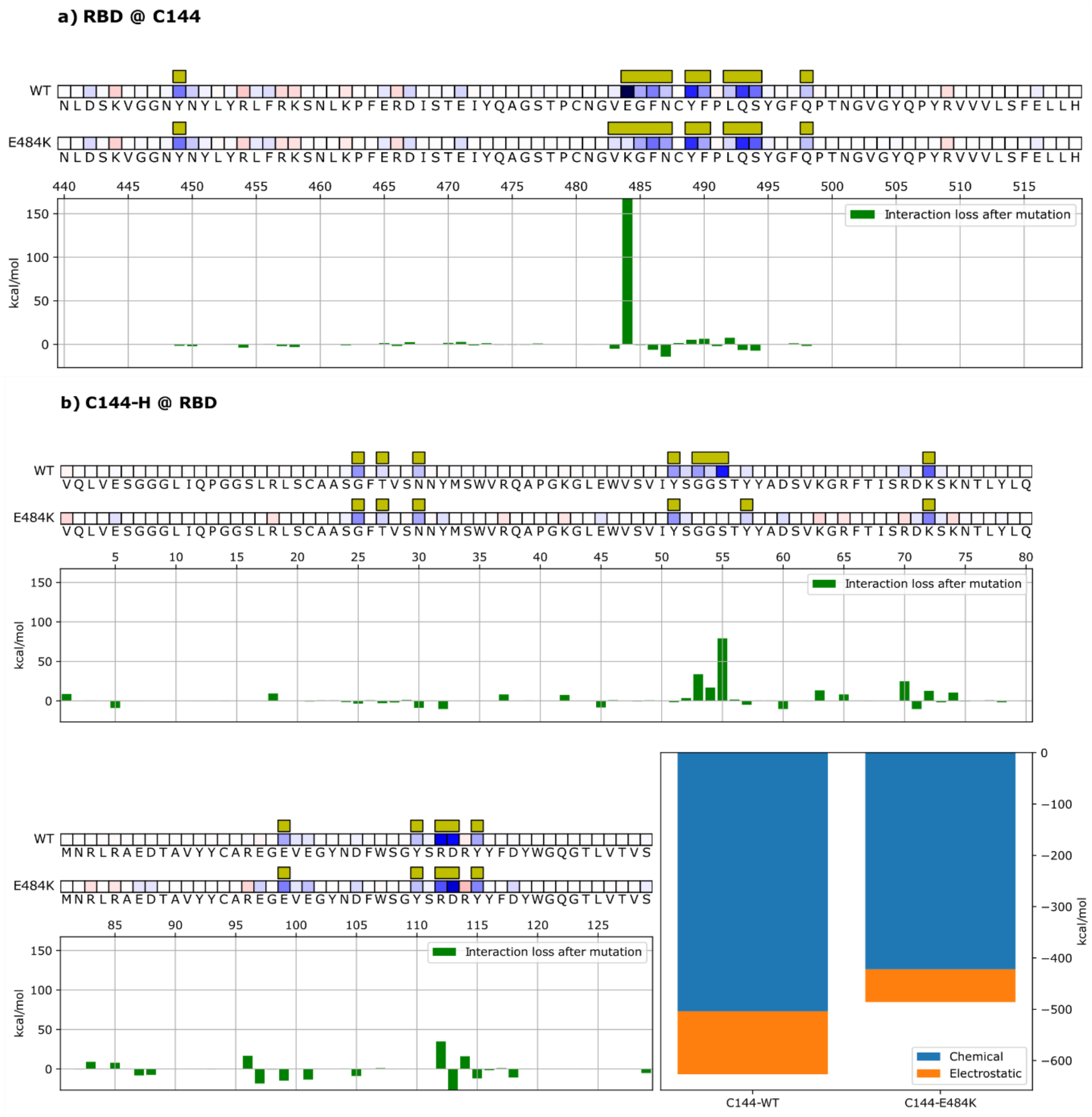

Fig S3. Mechanistic characterization of C144 binding to the Wuhan spike, and energetic changes as a result of the E484K spike mutation. Data are plotted on the spike primary structure (panel a) and on C144's (panel b), to compare the Wuhan spike (WT) and the mutated one (E484K). Amino acids are represented by the letters and numbered on the histogram's horizontal axis. Histograms underneath the sequences represent the relative change in binding energy of the E484K mutant relative to the Wuhan strain. The bar plots (bottom right) represent the chemical and electrostatic contributions in the binding of C144 to the WT and mutated spikes.

c)

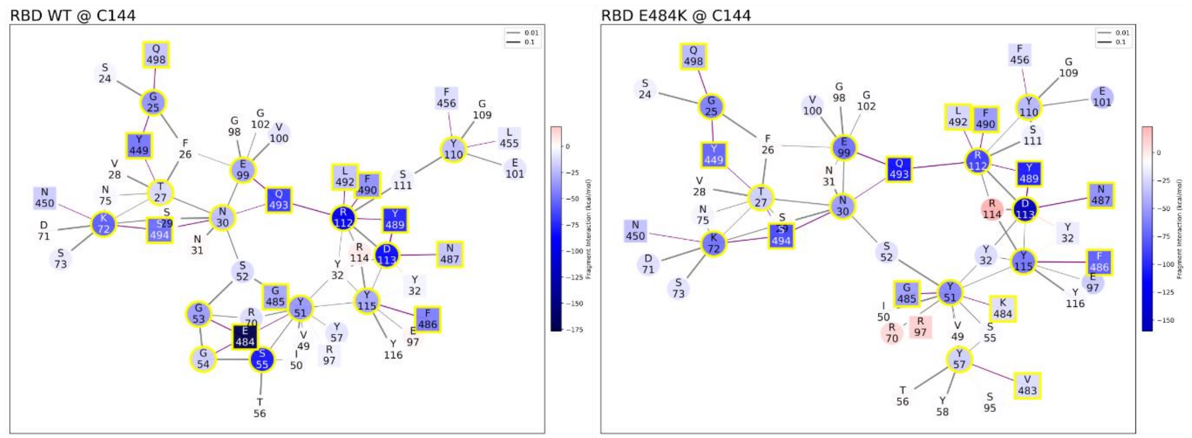

Fig S3. (cont.). Interaction networks (RBD WT @ C144 to the left, RBD E484K @ C144 to the right) at the bottom represent the interface residues and their coordinated interactors: squares are spike residues and circles C144's, their respective coloring is red for repulsive and blue for attractive energy, a yellow highlight represents interface residues. Bonds are purple when intermolecular or black when intramolecular. Interface residues are represented by red (repulsive) or blue (attractive) squares based on their effect to their counterpart and highlighted by yellow squares when at the binding interface.

The Beta variant Spike’s crystal structure can be decomposed into the sum of point-mutated virtual crystals derived from the Wuhan structure

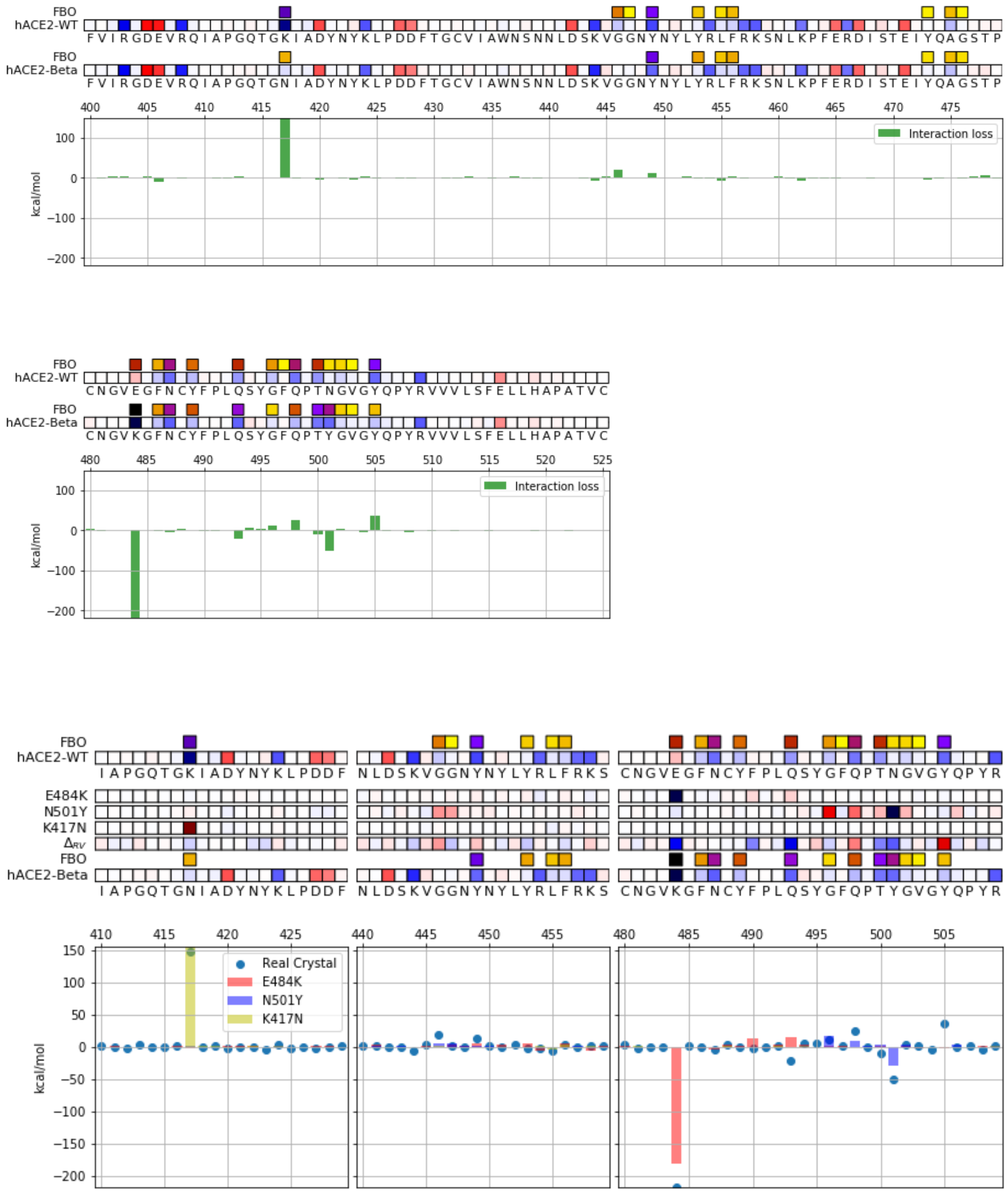

Fig S4. Spike-hACE2 interactions are consistent when comparing the calculated results between the experimentally characterized crystal and those obtained by virtually mutating the WT crystal. Top: comparison of contributions of spike amino acids between the WT strain and The Beta variant, when interacting with the hACE2 receptor. Bottom: The overall interactions with the Beta variant obtained from the experimentally characterized crystal can be adequately approximated as some of contributions when individual mutations are introduced virtually into the WT crystal.

The interaction energies of relevant residues are marginally affected by the implicit solvent

In light of the importance implicit solvent may have in the dynamics of binding, especially whenever electrostatic interactions play a substantial role, we verify its impact in the system under study. Results show that introducing an implicit solvent does not affect predictions.

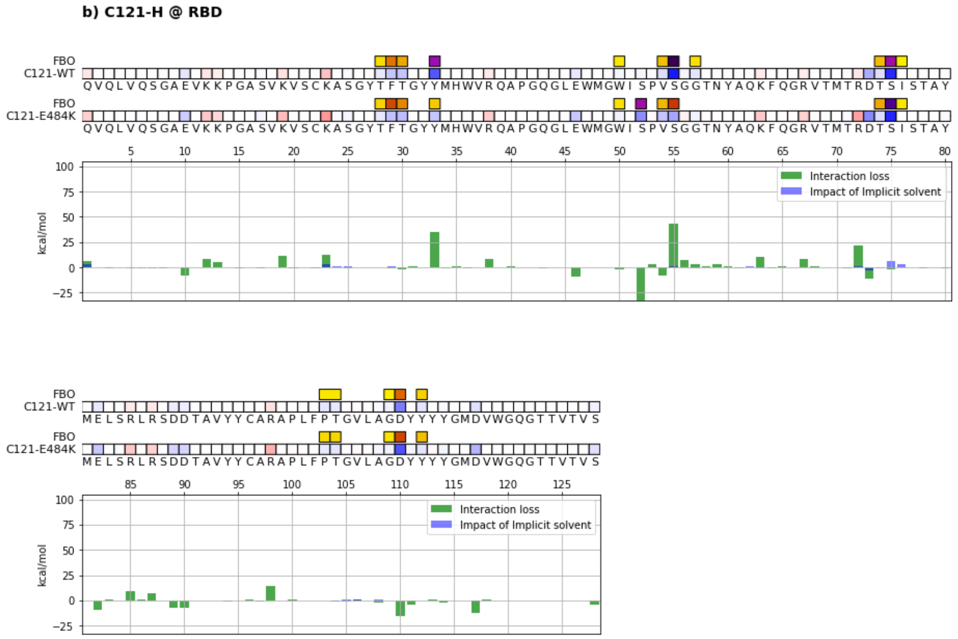

Fig S5. Mechanistic characterization of C121 binding to the Wuhan spike and E484K variant, and energetic changes as a result of addition of the E484K mutation. Data are plotted on C121structure to compare the Wuhan spike (WT) and the mutated one (E484K) effect on binding. Amino acids are represented by the letters and numbered on the histogram's horizontal axis. Histograms underneath the sequences represent the relative change in binding energy of the E484K mutant relative to the Wuhan strain. The bar plots (bottom right) represent the chemical and electrostatic contributions in the binding of C121 to the WT and mutated spikes. The impact of implicit solvent is highlighted by the blue bars, and is shown to be marginal.

Model comparison with preexisting experimental datasets on randomly generated libraries of hACE2 and viral spike mutants

The binding energies provided by our model can be compared, under reasonable assumptions on the structures employed, to the quantities obtained from experimental databases of libraries of the interaction between spike RBD mutants and hACE2 mutants, all available in the literature [8, 9]. While the comparison between these quantities is unconventional, as computational studies of protein-protein affinity involve in-depth analysis of structural and thermodynamic contributions

[58], results from both datasets are in general accordance (Fig S6).

We decompose the binding energy of the RBD with the ligand into per-residue contributions to describe the relative importance of each amino acid in the binding process. To see how these data compare with available experimental measurements, we generate a dataset of virtual point-mutations for the RBD-hACE2 system and compare the interaction energies with experimental affinity data obtained from existing high throughput random mutation experiments, which are available in the literature both for the Wuhan spike protein [9] and hACE2 [8]. Data are presented in Fig S6. As already pointed out in the main text, this comparison is unconventional, as computational studies of protein-protein affinity always include in-depth analysis of structural and thermodynamic contributions [58]. However, our simulation largely aligns with experimental results [8, 9, 35, 36]. The tested single point mutations do not lead to substantial structural rearrangement of the spike. Interestingly, the E484K mutation stands out as an outlier in both the experimental dataset and our simulation, exhibiting an overall improved binding to hACE2. However, most mutations of the RBD residues lead to decreased affinity, indicating that the viral Wuhan spike is overall well-adapted to bind hACE2.

One case where our model does not agree with experimental results is the case of mutation N501Y. We compared the structure we generated by point mutation along with the Beta crystal with the analysis of a molecular dynamics trajectory performed by Spinello et al. [44]. In the trajectory, conformational change, realized over 2 microseconds of simulation time, a hydrogen bond is formed between Tyr501 and Asp38. This feature is not present in the crystals we analyzed. The improvement in binding associated with mutation N501Y may indeed be less “RBD-Modular" than E484K, requiring significant conformational change in combination with other mutations to realize improved binding. To understand this mutation in detail, future studies based on our QM-CR approach should be combined with cutting edge molecular dynamics simulation.

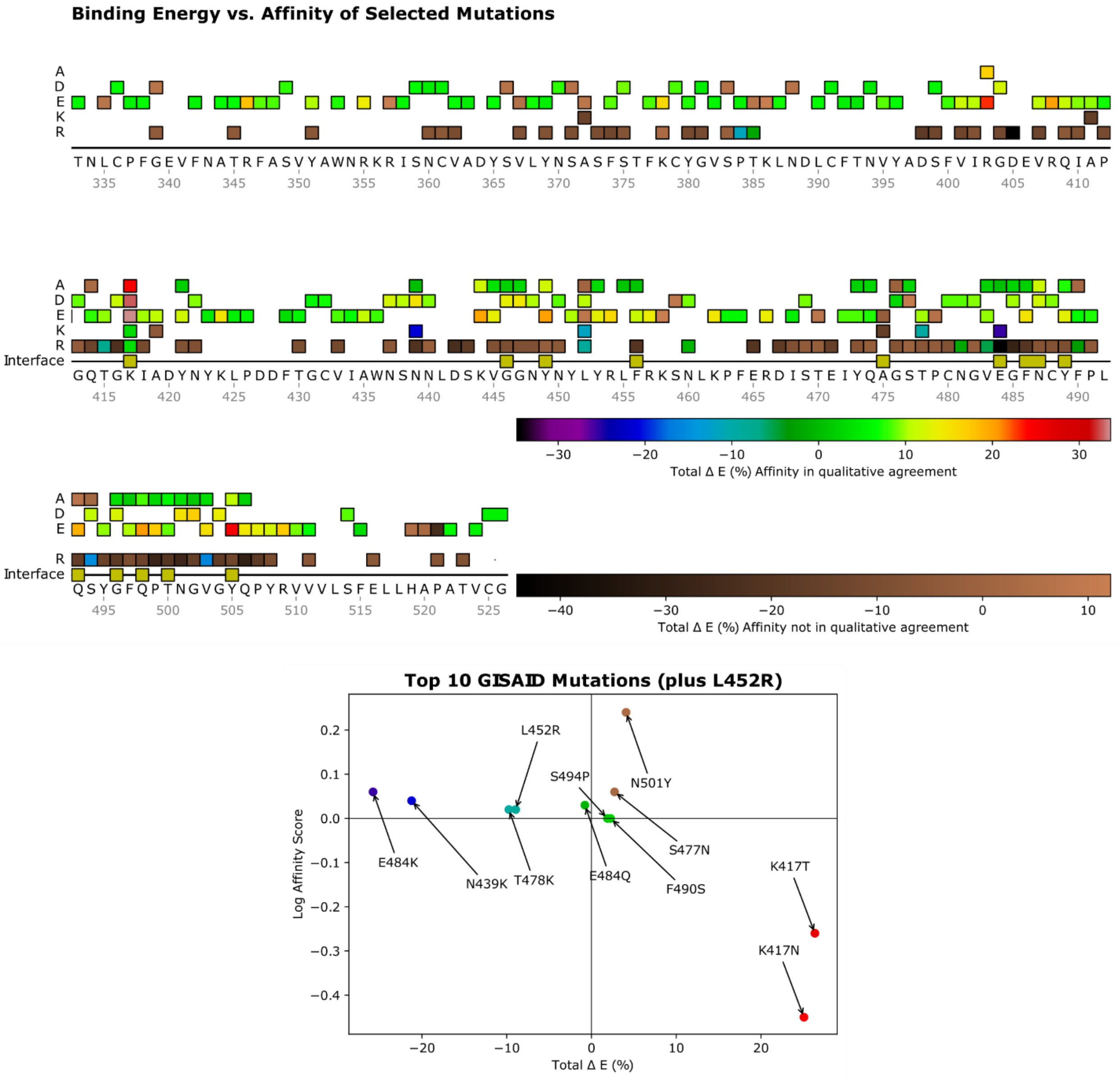

Fig S6. Comparison of QM-CR simulations of virtual structures with available *in vitro* binding performance of mutant libraries. Spike mutations are represented for their affinity binding score towards hACE2 [9]. Negative score means depletion of the affinity of mutated sequence. Virtual crystals have been generated *in silico* and the binding strength simulated using QM-CR. The percent difference in binding strength is then calculated with respect to Wuhan strain, negative value indicating improvement of the binding. If both the *in vitro* and the *in-silico* data represent a depletion or an enhancement, data are in qualitative agreement and colored with rainbow colors, from red to purple (error bars and uncertainties are not shown), and with copper colors otherwise. The *in vitro* and the *in silico* quantities are represented against each other (horizontal axis for the QM-CR binding, vertical axis for the affinity), by focusing on the top 10 GISAID [59] point-mutations (plus the L452R point-mutation) which are found in SARS-CoV-2 variants at the time of writing this contribution. The same color scheme is employed for the mutation represented in the sequence and for the plots. The two datasets have many points in accordance, more than 60% of the points being in qualitative agreement. Among all the tested mutations, E484K (purple point) emerges as the strongest binding performance in qualitative agreement with the experiment.

Details of the Fragmentation procedure

We here recall the main equations that define the energy decomposition schemes employed in the present work. We identify a system $\mathcal{S}$ from the set of its atomic positions. A QM calculation of the system $\mathcal{S}$ provides the density matrix $F[\mathcal{S}]$.

We associate the atomic positions of the system $\mathcal{S}$ to a set of ionic (electronic) charge densities $\rho^{\mathcal{S}}_{ion(H)}$, respectively. The expression of the total energy reads:

$$E[\mathcal{S}] = tr\left(\hat{H}_{K\mathcal{S}}[\mathcal{S}]\widehat{F_{\mathcal{S}}}\right) - E_H\left[\widehat{F_{\mathcal{S}}}\right] - tr\left(\hat{V}_{xc}\left[\widehat{F_{\mathcal{S}}}\right]\widehat{F_{\mathcal{S}}}\right) + E_{xc}\left[\widehat{F_{\mathcal{S}}}\right] + E_{ion}[\mathcal{S}]. \quad (1)$$

The DFT Hamiltonian is defined as $\hat{H}_{K\mathcal{S}} = -\frac{1}{2}\nabla^2 + \hat{\mathrm{V}}_H + \hat{\mathrm{V}}_{ion} + \hat{\mathrm{V}}_{xc}$ from the combination of the electrostatic potential provided by $\rho^{\mathcal{S}}_{tot} = \rho^{\mathcal{S}}_{ion} + \rho^{\mathcal{S}}_{H}$, including the exchange and correlation term.

When post-processing a QM DFT calculation, it is in theory possible to perform an analysis on an arbitrarily defined set of fragments. The challenge is to define those fragments in a chemically meaningful way, or, at least, to quantify the pertinence of a given fragmentation. To augment the choice of fragmentation scheme, in a previous study we introduced a measure of fragment quality called the purity indicator [33]. This measure is directly based on the density, being computed as the deviation from idempotency of the density matrix block associated with a given fragment.

The interactions of a system may be described in the same framework by computing a quasi-observable called the “Fragment Bond Order” as a measure of the off-diagonal contributions of the density matrix. As described in [33], the Purity Indicator and Fragment Bond Order are computed directly from the density matrix. The Purity Indicator and Fragment Bond Order together measure the competition between a fragment's internal and external interactions. This approach has recently been applied to generating graph views of proteins as well as to understand the interaction between proteins and solvent molecules [60].

Let us now suppose that our system is separated into two regions, which we call $\mathcal{F}$ and $\mathcal{G}$. We also assume that those two regions are associated with well-defined fragments, with associated fragment projection operators $\widehat{W}_{\mathcal{F},\mathcal{G}}$. We can then identify, for instance, $F[\mathcal{G}] = \widehat{W}_{\mathcal{G}} F[\mathcal{S}]\widehat{W}_{\mathcal{G}}$. The operators $F[\mathcal{F}]$ and $F[\mathcal{G}]$ are then defined as the diagonal projection of the full density $F[\mathcal{S}]$ of the assembly into the regions associated with the subsystems $\mathcal{F}$ and $\mathcal{G}$. We define by $\delta\widehat{F_{\mathcal{S}}}$ the off-diagonal term $\widehat{W}_{\mathcal{G}} F[\mathcal{S}]\widehat{W}_{\mathcal{F}} + \widehat{W}_{\mathcal{F}} F[\mathcal{S}]\widehat{W}_{\mathcal{G}}$. With these definitions, we can identify different terms contributing to the interaction of the fragments. Let us describe them separately.

- Electrostatic, long-range term: it is defined by the electrostatic interaction between the two fragments in their polarized state, i.e., the ground state of the assembly.

$$E_{el} = \left(\rho_{tot}^{\mathcal{F}} \middle| \rho_{tot}^{\mathcal{G}}\right) \qquad (1)$$

This is the only contribution which will have to be considered even when the $\mathcal{F}$ and $\mathcal{G}$ subsystems are spatially separated from each other. Such a term can be efficiently approximated by a sum of point-multipoles that are based on the description of the electron density. The electrostatic terms can be non-negligible even for fragments that are far apart, as the interaction which defines this term is long-range.

- Chemical/Contact, short-range term: it is defined by the trace of the block-off-diagonal part of the DFT Hamiltonian on the density of the total system. It reads:

$$E_{ct} = tr\left(\hat{H}_{K\mathcal{S}}[\mathcal{S}]\delta\widehat{F_{\mathcal{S}}}\right) = tr\left(\widehat{W_{\mathcal{F}}}\hat{H}_{K\mathcal{S}}[\mathcal{S}]\widehat{W_{\mathcal{G}}}\widehat{F_{\mathcal{S}}}\right) + tr\left(\widehat{W_{\mathcal{G}}}\hat{H}_{K\mathcal{S}}[\mathcal{S}]\widehat{W_{\mathcal{F}}}\widehat{F_{\mathcal{S}}}\right) \qquad (1)$$

This term is a "contact" term, non-zero only for fragments which lie nearby, as the Hamiltonian and the density matrix are both represented by sparse matrices. Also, its interpretation is associated with the energy the system would lose if both fragments were to be considered as pure. As the purity indicator is associated with the fragment valence, this term is associated with the energy of the "chemical bond"" between the fragments. For this reason, it is always associated with an attractive term, and its magnitude is directly correlated to the value of the fragment bond order. For compactness, dispersion van der Waals contributions, coming from D3 terms [49], are included in such a term, as the latter are also short-range. The chemical term is always stabilizing, in that it only contains attractive interactions.

We have verified that the sum of these terms correlates very well with the QM interaction energy between the subsystems $\mathcal{F}$ and $\mathcal{G}$, thereby proving that they capture the essential contributions of the interactions, in our analysis [45].

Both these terms can be decomposed in per-fragment contributions; through the decomposition, we obtain an indication of the relative contribution that each fragment (in our case, each residue) provides to the interaction terms between the two assemblies. We represent those contributions in a network of interactions determined by the FBO quantity. (Absolute) purity values below 0.05 (see previous work [33, 60] for a justification of this threshold) indicate that the fragments are sufficiently pure and thus represent a good decomposition of the system. For the systems employed in this work, most amino acids lie within this range, with some exceptions (mainly

Glycine residues) which reach a (yet acceptable) value of 0.07. For this reason, and to ease the discussion, we have preferred to stick with a usual fragmentation scheme based on amino acids.
